# The Current Role of Cardiopulmonary Exercise Test in the Diagnosis and Management of Pulmonary Hypertension

**DOI:** 10.3390/jcm12175465

**Published:** 2023-08-23

**Authors:** Beatrice Pezzuto, Piergiuseppe Agostoni

**Affiliations:** 1 Centro Cardiologico Monzino IRCCS, 20138 Milan, Italy; beatrice.pezzuto@cardiologicomonzino.it; 2 Department of Clinical Sciences and Community Health, University of Milan, 20122 Milan, Italy

**Keywords:** pulmonary hypertension, cardiopulmonary exercise test, prognosis

## Abstract

Pulmonary arterial hypertension (PAH) is a progressive disease with a poor prognosis if left untreated. Despite remarkable achievements in understanding disease pathophysiology, specific treatments, and therapeutic strategies, we are still far from a definitive cure for the disease, and numerous evidences have underlined the importance of early diagnosis and treatment to improve the prognosis. Cardiopulmonary exercise testing (CPET) is the gold standard for assessing functional capacity and evaluating the pathophysiological mechanisms underlying exercise limitation. As effort dyspnea is the earliest and one of the main clinical manifestations of PAH, CPET has been shown to provide valid support in early detection, differential diagnosis, and prognostic stratification of PAH patients, being a useful tool in both the first approach to patients and follow-up. The purpose of this review is to present the current applications of CPET in pulmonary hypertension and to propose possible future utilization to be further investigated.

## 1. Introduction

Pulmonary arterial hypertension (PAH) is a rare disease characterized by an increase in pulmonary vascular resistance (PVR) due to remodeling, fibrosis, and thrombosis in situ of the pulmonary arterioles, with consequent pressure overload and right heart failure [1]. PAH is a progressive disease with a poor prognosis which, if left untreated, inevitably evolves to death [2].

Several studies have shown that early diagnosis and treatment are essential for changing the course of the disease and improving the prognosis [3,4,5]. The main early symptom of PAH is effort dyspnea, which is due to the inability of the right ventricle, burdened by an excessive afterload, to maintain a cardiac output adequate to the increased metabolic needs of the organism during exercise. Only later, overt congestive heart failure develops, with low cardiac output at rest and fluid retention [6]. Consequently, identifying the presence of PAH from exercise-induced dyspnea is paramount for an efficacious treatment.

Cardiopulmonary exercise testing (CPET) is the gold standard method to assess functional capacity, the presence of exercise limitation, and its causes, including PAH [7].

Through the analysis of ventilation and exhaled gases, CPET provides information about the cardiovascular, respiratory, metabolic, and muscular response to physical effort, investigating the pathophysiological mechanisms underlying the functional limitation, but also providing parameters of fundamental prognostic importance.

The latest ESC/ERS guidelines on pulmonary hypertension (PH) have emphasized the role of CPET in the management of the disease, both in the initial diagnostic phase and in the follow-up [8].

The purpose of this review is to discuss the currently recognized role of CPET in the management of patients with PH and to propose perspectives for possible future applications.

## 2. Pathophysiological Model of PAH and Causes of Effort Dyspnea in Affected Patients

The pathophysiological model of PAH is an afterload mismatch: the pulmonary arterial obstructive vasculopathy, which constitutes the first step in the pathogenetic process of the disease, induces an increase in pulmonary vascular resistance (PVR) and mean pulmonary arterial pressure (mPAP), leading to increased right ventricular (RV) afterload. In the early stages of the disease, the RV tries to adapt to the increased afterload by increasing contractility, with or without a minimum increase in the right heart size. In the more advanced stages, the RV fails to remain coupled to the increased afterload, with consequent progressive right heart dilation and RV systolic dysfunction; moreover, the development of myocardial fibrosis and sarcomeric stiffening cause diastolic dysfunction [9]. The results of such morphofunctional changes are a reduction in RV output, an increase in RV filling pressures, and reduced left ventricular (LV) filling, with altered ventricular interaction and reduced systemic pressure [2,9,10,11].

The RV’s ability to adapt to increased afterload (RV-arterial coupling) is the main determinant of symptoms, clinical status, and prognosis of affected patients [2]. Even at the earlier stages of the disease, increasing pulmonary blood flow during exercise, which in healthy subjects is normally accommodated by vascular distention and new vessels recruitment to maintain low PVR, in PAH patients leads to a further increase in PAP, as a consequence of a pulmonary arteriolar district obliterated and not distendable due to vasculopathy, and by the absence or near-absence of recruitable vessels [12]. A ventricular–arterial coupling inadequate to face this progressive elevation in the afterload limits the ability of the RV to augment the stroke volume [13], and the increase in cardiac output (CO) during exercise strongly depends on the heart rate (HR) [14]. Thus, right ventricular dysfunction is the first cause of effort dyspnea in PAH patients, as CO fails to increase during exercise due to a further rise in mPAP and RV-arterial uncoupling, with a consequent inadequate tissue oxygen delivery leading to early lactic acidosis [13,15,16].

Although RV dysfunction is the primary determinant of the clinical manifestations of the disease, there are other important causes of exertional dyspnea in PAH. Among those, the first is ventilation–perfusion mismatch due to the presence of reduced perfusion of well-ventilated alveoli, which leads to an increase in physiologic dead space (VD) to tidal volume (VT) ratio (VD/VT). The increased VD/VT is one, but not the only, cause of the increased ventilatory response to physical activity, which is the ultimate cause of the inefficient ventilation observed in PAH [17].

Another cause of exertional dyspnea in PAH patients is the increased chemoreflex response, leading to abnormalities in arterial blood gases and, specifically, arterial hypocapnia. Altered chemosensitivity in PAH may be explained by several mechanisms: activation of a sympathetic response to low CO as a consequence of RV failure (similar to left ventricular failure); an increase of catecholamines; the elevation of arterial potassium levels; progressive lactacidemia; enhanced RV distension; higher pulmonary intra-vascular pressures; hypoxaemia and increased oscillations in pH [18]; an increase in chemosensitivity for oxygen (O_2_) and carbon dioxide (CO_2_) [19], possibly due to low O_2_ partial pressure; unbuffered acidosis [20,21].

A further cause of effort dyspnea is hypoxia, which can be secondary to several mechanisms: low mixed venous O_2_ saturation, high dead space, low alveolar–arterial diffusion, right-to-left shunt through either a patent foramen ovale or intrapulmonary shunt. Hypoxaemia leads to the early occurrence of lactic acidosis and stimulates the carotid bodies to augment the ventilatory drive, contributing to exercise hyperventilation [22].

Finally, abnormal respiratory mechanics and peripheral muscle dysfunction may also contribute to exercise dyspnea in PAH. Several mechanical ventilatory abnormalities have been shown in PAH patients, such as restrictive ventilatory patterns [23], peripheral airflow limitation, and resting lung hyperinflation [24,25], while peripheral muscle dysfunction in PAH has been related to low CO, vasoconstriction, deconditioning, and altered skeletal muscle microcirculation [26].

## 3. Characteristics of CPET in PAH Patients

CPET of PAH patients can have three different categories of abnormalities: ventilatory abnormalities, gas exchange abnormalities, and cardiovascular abnormalities.

**Figure 1:**
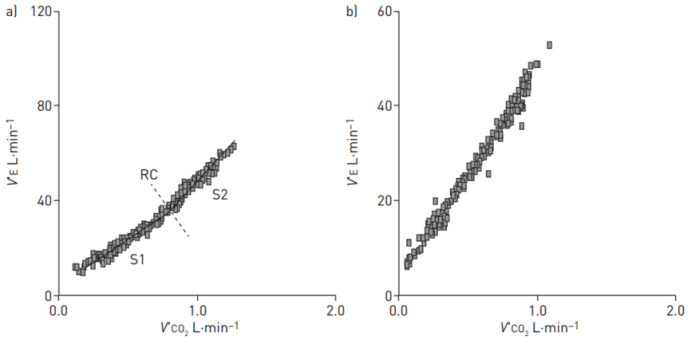
The minute ventilation (V_E_)/carbon dioxide production (VCO_2_) relationship slope in two pulmonary arterial hypertension patients. In (**a**), the physiological behavior of ventilation during exercise is preserved in spite of hyperventilation, such that two slopes can be recognized around the respiratory compensation (RC) point (slope 1 (S1) = 40.4 and slope 2 (S2) = 55.1). In (**b**), a single slope characterizes the V_E_/VCO_2_ relationship. Reproduced with permission of the © ERS 2023: *European Respiratory Review* 27 (148), 170134; DOI: 10.1183/16000617.0134-2017 Published 2 May 2018 [28].

Abnormalities of ventilatory function are mainly due to the obstructive pulmonary arterial vasculopathy characteristic of the disease. Indeed, PAH patients present with an increase in dead space ventilation during exercise, expressed by VD/VT ratio > 30%, while in normal subjects, it decreases [19,27]. The increased ventilatory response to exercise is reflected in high ventilatory equivalents for carbon dioxide and oxygen (V_E_/VCO_2_ and V_E_/VO_2_ ratio, respectively). Specifically, in an exercise with a ramp protocol, both the V_E_/VCO_2_ and V_E_/VO_2_ ratios do not show the normal reduction observed at the beginning of the exercise, but are flat in case of moderate PAH or increase in case of severe PAH. Similarly, the V_E_/VCO_2_ relationship slope is increased [17]. In some patients, the slope is so steep that it is no longer possible to identify the physiological upward deflection normally observed around the point of respiratory compensation (Figure 1) [28].

The elevated slope of the V_E_/VCO_2_ relationship is associated with a null or even negative *Y*-axis (ventilation) intercept. This finding, which is unique for PAH, is due to a progressive relevant increase of VD during exercise [29,30]. Of note, the modeling of increased VD and exercise was proposed by Gargiulo et al. in 2005 [29], who evaluated a human model of respiratory impairment in 10 heart failure (HF) patients and in 10 healthy subjects tested with a progressive workload exercise protocol with different added dead space. It was found that, in both patients and normal subjects, the increased serial dead space upshifts the V_E_/VCO_2_ slope, suggesting that V_E_-axis is related to dead space ventilation, thus allowing a noninvasive estimation of dead space volume. A subsequent multicenter retrospective study by Apostolo and colleagues [30] analyzed the V_E_-VCO_2_ relationship both as the slope and intercept in HF, HF-chronic obstructive pulmonary disease (COPD), COPD, PAH patients, and healthy subjects. The V_E_/VCO_2_ slope resulted highest and lowest in PAH and healthy subjects, respectively. No slope differences were observed among HF, HF-COPD, and COPD patients; on the other hand, the *Y*-axis intercept (V_E int_) was higher in HF-COPD and COPD compared to HF, PAH, and controls, and a value of V_E int_ ≥ 4.07 L/min identified patients with a high probability of having COPD or HF-COPD. These studies, in particular, suggest that CPET parameters can be of support in the evaluation of patients with PH and comorbidities, helping to understand which of the multiple mechanisms involved is the one most responsible for the symptomatology.

**Figure 2:**
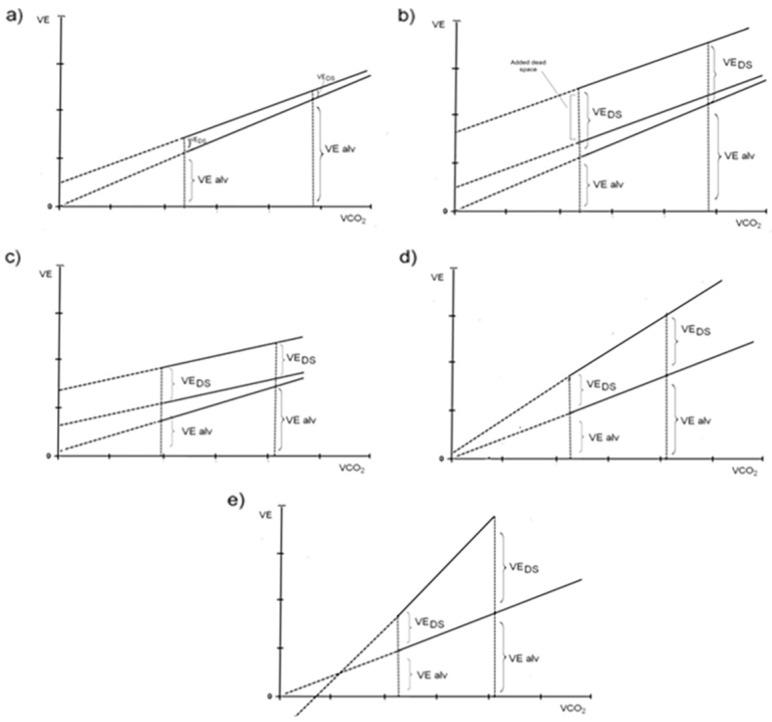
A schematic representation of the V_E_/VCO_2_ slope in a ramp incremental exercise test. Panel (**a**), normal subject—Lower line: alveolar ventilation (V_E alv_) vs. VCO_2_ up to the isocapnic buffering period. Upper line: total ventilation (V_E alv_ + naturally-occurring dead space V_E DS natural_) vs. VCO_2_. Dotted lines: extrapolation to the *Y* axis (V_E_ intercepts) for both V_E alv_ and total ventilation. Panel (**b**), normal subject with added external dead space (DS)—In addition to the lines depicted in panel (**a**), highest line: total ventilation plus the added DS (V_E alv_ + V_E DS natural_ + V_E DS added_) vs. VCO_2_. Panel (**c**), patient with moderate-to severe COPD—V_E alv_ vs. VCO_2_ relationship as in panels (**a**,**b**). Panel (**d**), patient with moderate heart failure—V_E alv_ vs. VCO_2_ as in panels (**a**–**c**). V_E DS_ shows a moderate increase resulting in a slight slope increase with an extrapolation to the *Y* axis (intercept) close to 0. Panel (**e**), patient with severe heart failure or pulmonary arterial hypertension—V_E alv_ vs. VCO_2_ relationship as in upper panels. V_E DS_ increases during exercise. The extrapolation to the *Y* axis (intercept) of total ventilation now has a negative value (From Apostolo A et al., *Int. J. Cardiol*. 2015, 189, 134–140 [30], with permission).

The physiological interpretation proposed by Apostolo et al. [30] is of utmost importance to understand the V_E_/VCO_2_ slope behavior and the Y-intercept in patients with different cardiovascular diseases. Indeed, the Y-intercept is mainly affected by exercise-induced changes in DS (Figure 2): in normal subjects, V_E int_ represents exercise VD if the latter remains unchanged, or it would be slightly higher if VD decreases during exercise, while if VD is increased at rest but remains stable during exercise, V_E int_ will be increased with minimal changes in the V_E_/VCO_2_ slope, as in HF patients with an external added dead space occurs. In patients with COPD, there is a substantial increase in V_E int_ due to a naturally-occurring high VD at the beginning of exercise, which compounds to a high alveolar ventilation (V_E alv_) driven by a shallower slope. In HF patients, the V_E_/VCO_2_ slope is normal or slightly increased, while V_E int_ is positive. Alternatively, when progressive increases in VD during exercise are accompanied by increases in ventilatory drive, this leads to very steep V_E_/VCO_2_ slopes, as in PAH patients, where the V_E int_ becomes negative.

The ventilation-perfusion mismatch and the altered VD/VT ratio are responsible for the second category of CPET alterations in PAH, that is gas exchange abnormalities, at first the frequently observed reduction of end-tidal pressure of CO_2_ (P_ET_CO_2_) at rest and during exercise, as the ventilatory inefficiency causes the dilution of P_ET_CO_2_ [31]. Moreover, the arterial partial pressure of CO_2_ (PaCO_2_) is low due to the reflex increase of ventilation. Indeed VD/VT abnormalities cause P_ET_CO_2_ reduction but do not cause PaCO_2_ reduction. The arterial to end-tidal carbon dioxide tension difference P(a-_ET_)CO_2_ is positive at rest and increases during effort in patients with PAH, while in healthy subjects is reduced and often negative at peak exercise [32]. Finally, the arterial to alveolar oxygen tension difference P(A-a)O_2_ is higher than normal in PAH patients [15]. Of note, P(a-_ET_)CO_2_ and P(A-a)O_2_ require direct measurement of arterial blood gases during exercise. It must be underlined that a few studies suggest reporting the P(A-a)O_2_ gradient normalized for VO_2_ [33,34].

The third category of CPET abnormalities in PAH is related to impaired cardiovascular function. Interestingly, although RV dysfunction is the main determinant of clinical status and prognosis of PAH patients, and the CPET abnormalities related to it are often very marked and have a significant prognostic impact, these aspects are not peculiar to patients with pulmonary vascular disease. These abnormalities include reduced oxygen consumption (VO_2_) at exercise peak, anaerobic threshold (AT), VO_2_ to work rate ratio (VO_2_/work slope), peak HR, and O_2_ pulse (VO_2_/HR) [35,36,37]. In particular, the VO_2_/work slope is often reduced in PAH patients; moreover, in the most severe PAH cases, it presents a deflection at the AT as a consequence of the greater dependence on anaerobic metabolism due to poor cardiac output and impaired peripheral muscle oxygen use [26,35].

The O_2_ pulse is usually reduced in PAH patients, and its kinetics are characterized by a blunted or flattened slope, so that the cardiac output changes are mainly related to the HR increase [13,35].

Peak HR is also reduced as the chronotropic response is impaired. However, the HR/VO_2_ slope is increased and shifted upward [35,38], again suggesting that cardiac output increase is due to HR increase and not to stroke volume increase [13]. HR behavior during exercise differentiates patients with PAH from those with HF: in fact, at the same level of functional limitation and of peak VO_2_, PAH patients have a smaller SV response, which is compensated by an increased HR response and, thus, a steeper slope of HR relating to oxygen uptake compared with left HF patients [36]. After exercise, the rate of recovery in HR is abnormally prolonged in patients with PAH, and similar to that seen in left ventricular failure [38].

Exercise oscillatory ventilation (EOV) or exercise oscillatory breathing (EOB), is a ventilation abnormality frequently observed in patients with severe cardiovascular diseases. As regards PH, EOV is frequently observed in post-capillary PH [39], but not in PAH [40]. Several are the hypothesis of this different behavior: (a) increased pulmonary wedge pressure in post capillary PH only, which leads to the development of oscillatory respiration in HF but not in PAH patients [41,42], (b) an upstream “protective” effect by the pre-capillary component in PH-LHD, limiting the afferent input implicated in the genesis of oscillatory ventilation [43], (c) the same reflex mechanism underlying exercise-induced hyperventilation which, centrally interacting with chemoreflex and leftward shifting the CO_2_ threshold [43,44], overrides and stabilizes the ventilatory oscillations.

In addition to defining the main characteristics of patients with significant pulmonary vascular disease, CPET has also proved useful in detecting differences in the response to exercise between specific forms of PAH and in understanding the underlying pathophysiological mechanisms. For example, a study evaluating 167 PH [45], 57 grown-up congenital heart disease (GUCH) and 110 non-GUCH patients, showed that in PAH-GUCH patients PAP and V_E_/VCO_2_ slope were higher compared to non-GUCH PH patients, while pulmonary blood flow and peak VO_2_ were lower. After matching patients for gender and peak VO_2_, PAP and V_E_/VCO_2_ slope remained higher in GUCH patients. These results suggested that long-term adaptation to high pulmonary pressure, hypoxia and low pulmonary blood flow, and persisting shunt could, at least partially, preserve exercise performance of GUCH PAH patients.

## 4. Role of CPET in PH Diagnostic Phase and PH Differential Diagnosis

The latest ESC/ERS guidelines [8] have emphasized the role of CPET in the diagnostic phase of PH. In fact, it is included among the examinations to be considered in symptomatic patients with intermediate echocardiographic probability of PH, to further determine the likelihood of PH, in symptomatic patients with systemic sclerosis (SSc) o other connective tissue disease (CTD), to aid decisions to perform right heart catheterization (RHC), in symptomatic patients with mismatched perfusion lung defects beyond 3 months of anticoagulation for acute pulmonary embolism, in the suspicion of chronic thromboembolic pulmonary hypertension (CTEPH), and in symptomatic patients with portal hypertension or HIV, to screen for PAH.

Indeed, as effort dyspnea and exercise intolerance characteristic of the early stage of PAH are aspecific symptoms common to several other diseases, CPET is a valuable tool in noninvasively identifying patients more likely to have PH, or alternatively, to suggest other kinds of pathophysiological mechanisms underlying the symptomatology. Moreover, the value of V_E_/VCO_2_ slope and the presence of EOV are included among the parameters suggested for patient phenotyping and to assess the likelihood of left heart disease as the cause of PH [8].

Some CPET alterations in PAH patients are peculiar to the disease, being highly suggestive of pulmonary vascular dysfunction. In 2005, Yasunobu et al. [31] analyzed a population of both normal subjects and patients with PAH and showed that the end-tidal pressure of CO_2_ (P_ET_CO_2_) and the ventilatory equivalent of CO_2_ (V_E_/CO_2_) at AT presented a hyperbolic relationship, grading the likelihood of pulmonary vasculopathy accounting for exertional dyspnea of unknown cause: the higher the degree of impairment of both parameters (that is the higher the V_E_/VCO_2_, the lower the P_ET_CO_2_), the higher the likelihood to have PH, while with both parameters normal, the diagnosis of PH is very unlikely.

The relationship between P_ET_CO_2_ and V_E_/CO_2_ in idiopathic PAH as a tool to assess the likelihood of PAH was confirmed by a subsequent study by Valli et al. [46], performed both on a treadmill and cycle ergometer.

Moreover, several studies have highlighted the role of CPET in identifying PH forms of the first group of the clinical classification other than idiopathic PAH.

Among those, Leveneziana et al. [47] found that 8 patients with pulmonary veno-occlusive disease (PVOD) had significantly lower P_ET_CO_2_ values, higher V_E_, and consequently higher V_E_/VCO_2_ slopes compared to 16-matched patients with PAH. A subsequent larger study [48] on 23 PVOD patients and a control group of 52 PAH patients confirmed higher V_E_/VCO_2_ slope and V_E_/CO_2_ at the AT values in PVOD vs. PAH; moreover, it showed a lower peak VO_2_ and earlier AT, and founded a predictive power for PVOD diagnosis for % predicted VO_2_ and V_E_/VCO_2_ slope in combination with PVR.

In 2016, Dumitrescu et al. [49] prospectively evaluated 173 consecutive multicentric patients with systemic sclerosis without known PAH, but with clinical suspicion of PAH, having undergone CPET and RHC. Peak VO_2_ and V_E_/VCO_2_ showed the highest correlations with PAP, transpulmonary pressure gradient, and PVR. Several parameters showed high sensitivity and specificity for PAH detection by ROC analysis; however, peak VO_2_ showed the highest diagnostic accuracy, with a sensitivity of 87.5% and specificity of 74.8% at a threshold of 13.8 mL/min/kg, while a value >18.7 mL/min/kg excluded PAH in all patients, resulting as the best discriminator between patients with and without PAH. Ventilatory efficiency parameters (V_E_/VCO_2_, P_ET_CO_2_) resulted in being inferior to peak VO_2_ in detecting SSc-associated PAH, and only a nadir V_E_/VCO_2_ > 45.4 was strongly predictive of PAH (positive predictive value 1.0). This study suggested the role of CPET in reducing unnecessary RHC procedures in SSc patients with PAH suspicion.

In a subsequent study, Santaniello and colleagues [50] screened 314 consecutive SSc patients with the DETECT algorithm, and positive subjects were referred for CPET before the execution of RHC. The DETECT algorithm [51] has been developed to identify SSc patients at risk for PAH, and involves two steps in succession: step 1 evaluates six clinical parameters (telangiectasias, anticentromere B antibodies positivity, BNP and uric acid increase, abnormal forced vital capacity/lung diffusion capacity for carbon monoxide (FVC/DLCO) ratio, and right axis deviation at ECG), whose cumulative score should reach a cut-off threshold to refer to echocardiography; in step 2, the step 1 prediction score and two echocardiographic variables, the tricuspid regurgitation velocity and right atrial area, determine the referral to RHC. However, the DETECT algorithm yields high sensitivity and a positive predictive value but low specificity. The predictive performance of CPET on top of DETECT was evaluated and internally validated by Santaniello via bootstrap replicates, founding that, within CPET variables, V_E_/VCO_2_ slope had the best performance to predict PAH at right-heart catheterization, with a specificity of 0.778 (0.714–0.846) and a positive predictive value of 0.636 (0.556–0.750).

In a more recent study [52] on 131 patients who underwent a CPET (112 CTD including SSc, overlap syndrome, mixed and undifferentiated CTD, 8 CTD-PAH, 11 PH of different etiology), among CPET parameters, peak VO_2_, V_E_/VCO_2_ slope, and P_ET_CO_2_ showed the best diagnostic performance for PAH, and were comparable among CTDs-PAH and PH of different etiology. The diagnostic performance was even improved by creating a composite score that included all three parameters identified.

CPET has also been tested in CTD other than SSc. In a study [53] on 21 systemic lupus erythematosus associated PAH (SLE PAH) patients and an equal number of IPAH patients and controls, peak VO_2_, P_ET_CO_2_, and peak O_2_ pulse were lower in SLE PAH than IPAH and controls, with the relationship of VO_2_ to V_E_ (that is the oxygen uptake efficiency slope (OUES)), being lower during all stages of exercise in SLE PAH. The peak O_2_ pulse and VO_2_ at AT in SLE PAH and IPAH were low, and significant difference between SLE PAH and IPAH was seen. PVR correlated with the lowest V_E_/VCO_2_, O_2_ pulse, peak P_ET_CO_2_, and OUE in SLE PAH patients.

Finally, CPET has also been extensively investigated in the other groups of PH clinical classification (Table 1).

In 2013, McCabe et al. [54] evaluated 15 patients with CTEPH and 15 with chronic thromboembolic disease without PH (CTED), all diagnosed after pulmonary embolism (PE) and having undergone CT pulmonary angiography, CPET and RHC, with 10 control subjects undergoing CPET. Peak exercise VD/VT, alveolar-arterial oxygen gradient, HR/VO_2_ slope, V_E_/VCO_2_ slope, V_E_/VCO_2_ at the AT, and P_ET_CO_2_ at AT discriminated between CTED and CTEPH groups, and VD/VT showed good correlation with mPAP. In a multivariate model comparison of VD/VT, alveolar–arterial gradient and V_E_/VCO_2_ at AT with serum NTpro-BNP and HR/VO_2_ slope, only VD/VT retained a significant predictive effect. Peak exercise VD/VT > 45% had a sensitivity of 92% and specificity of 83% in predicting a diagnosis of CTEPH, while a peak exercise alveolar–arterial oxygen gradient >32 mmHg had an equivalent sensitivity of 92% and specificity of 67%. This study highlighted the role of CPET in providing diagnostic insight into patients suffering persistent symptoms after acute PE and suggested a potential role of CPET in supporting the diagnosis of CTEPH and CTED.

Regarding the forms of IP associated with left heart disease, in a first retrospective analysis [39] on 293 prospectively collected HF subjects who had undergone Doppler echocardiography and CPET, a V_E_/VCO_2_ slope ≥ 36 was the best predictor of a systolic pulmonary arterial pressure ≥ 40 mmHg, and peak P_ET_CO_2_ ≤ 34 mmHg and the presence of EOV added significant diagnostic value.

In a subsequent retrospective study Caravita et al. [43] investigated the cardiorespiratory profile during exercise in isolated post-capillary PH (IpcPH), combined post-capillary PH (CpcPH) and idiopathic/heritable PAH, founding a stepwise increase of exercise-induced hyperventilation and a reduction of the occurrence of exercise oscillatory breathing (EOB) passing from IpcPH to CpcPH to PAH. Indeed, while the exercise in PAH patients was characterized by exercise-induced desaturation and exercise hyperventilation, with very low P_ET_CO_2_ and very high ventilatory equivalent for CO_2_, IpcPH population showed a high prevalence of EOB and a modest increase in exercise-induced hyperventilation, while CpcPH population behaved as in between IpcPH and PAH, with an intermediate pattern of ventilatory control derangement. Some correlations were found between markers of pulmonary vascular disease (such as diastolic pulmonary gradient and PVR) and V_E_/VCO_2_ slope, V_E_/VCO_2_ at the anaerobic threshold, and P_ET_CO_2_ at the anaerobic threshold.

Another study [55] on 90 subjects with dilated cardiomyopathy (DCM) that had undergone cardiac catheterization and CPET found that a peak VO_2_% predicted value of 52.5% was the best predictor of an mPAP ≥ 25 mmHg in the ROC analysis, while in the multivariate analysis, the same parameter was the only significant independent predictor of PH.

CPET has been shown to be a significant tool to support the detection of PH also in lung disease. In a population of 47 COPD patients GOLD II to IV divided into three groups based on mPAP, Boerrigter and colleagues [56] found that patients with moderate PH (mPAP 25–39 mmHg at RHC) presented only a mild alteration of V_E_/VCO_2_ slope and P_ET_CO_2_ compared with patients with no PH (mPAP < 25 mmHg), while patients with severe PH (mPAP > 40 mmHg) presented a much higher V_E_/VCO_2_ slope and lower P_ET_CO_2_ compared to both other study patients groups, but also a lower mixed venous O_2_ saturation, preserved breathing reserve and absence of CO_2_ increase during exercise.

A subsequent study [57] showed that among 38 patients with idiopathic pulmonary fibrosis (IPF), a V_E_/VCO_2_ > 45 at the AT allowed significant differentiation between subjects with and without PH (PH estimated with sPAP ≥ 40 mmHg); moreover, patients with a V_E_/VCO_2_ > 45 at the AT had a significantly lower survival compared to those with a V_E_/VCO_2_ ≤ 45, whereas sPAP did not allow survival prediction. Another study [58] showed that peak VO_2_ and V_E_/VCO_2_ slope differed significantly between IPF patients with and without PH, and survival was best predicted by the presence of PH, followed by peak VO_2_.

A more recent study found that alveolar–arterial O_2_ difference at peak exercise, V_E_/VCO_2_ slope, peak P(a-ET)CO_2_, and peak VO_2_ were parameters that had high sensitivity, and when combined, a high specificity to detect PH in patients with combined pulmonary fibrosis and emphysema (CPFE) [59].

## 5. Role of CPET in Staging Disease Severity

Several studies have shown the correlation between CPET parameters and PAH severity. In 2001, Sun and colleagues [35], in a retrospective analysis of 53 primitive PH (PPH) patients that had undergone RHC and cycle ergometer CPET, found consistent reductions in peak VO_2_, anaerobic threshold, peak O_2_ pulse and rate of increase in VO_2_; moreover, NYHA class correlated with the above-altered parameters.

In 2005, Yasunobu et al. [31], in a population of 52 PPH patients that had undergone CPET and RHC, found that the % of predicted peak VO_2_ correlated significantly with mPAP and P_ET_CO_2_ values at rest, while anaerobic threshold and peak VO_2_ were proportionately reduced as the percentage of predicted peak VO_2_ decreased. In addition, P_ET_CO_2_ values at rest, AT, and peak VO_2_ were reduced as mPAP increased. Moreover, they described the typical behavior of P_ET_CO_2_ in PH patients (decreasing from rest to AT and subsequently increasing at the start of recovery) compared to normal subjects (increasing from rest to AT and decreasing at the start of recovery).

**Figure 3:**
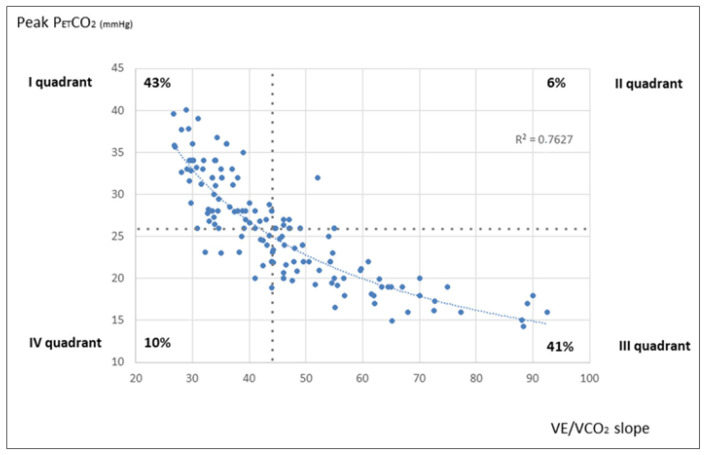
Scatterplot of the V_E_/VCO_2_ slope versus peak P_ET_CO_2_ (from Pezzuto et al., *Pulm. Circul.* 2022, 12, e12044 [60], with permission). P_ET_CO_2_: end-tidal carbon dioxide pressure; V_E_/VCO_2_ slope: ventilation to carbon dioxide production slope.

A more recent study [60] on 144 retrospective PAH patients performing CPET and RHC found that V_E_/VCO_2_ slope and peak P_ET_CO_2_ significantly varied in mPAP and PVR tertiles, while the peak VO_2_ and O_2_ pulse also varied in cardiac index and right atrial pressure tertiles. P_ET_CO_2_ versus V_E_/VCO_2_ slope showed a strong hyperbolic relationship, and patients with peak P_ET_CO_2_ > median (26 mmHg) and V_E_/VCO_2_ slope < median (44) presented lower mPAP and PVR than patients with peak P_ET_CO_2_ < median and V_E_/VCO_2_ slope > median (Figure 3).

## 6. Role of CPET in Prognostic Evaluation and Follow-Up of PAH

Previous ESC/ERS guidelines on PH [61] identified three groups of risk characterized by three colors, green (low risk), yellow (moderate risk), and red (high risk), based on several parameters, including peak VO_2_ and V_E_/VCO_2_ slope. The latest ESC/ERS guidelines [8] have confirmed the importance of risk stratification in patients with PAH, both at the time of the initial evaluation in order to choose the right therapeutic strategy and during the follow-up. According to guidelines, CPET has a well-recognized prognostic role at the time of baseline evaluation; in particular, peak VO_2_ values > 15 mL/kg/min and V_E_/VCO_2_ slope < 36 are associated with low risk (1 year mortality < 5%), 11–15 mL/kg/min and 36–44 respectively with intermediate risk (mortality at 1 year 5–20%), while peak VO_2_ < 11 mL/kg/min and >V_E_/VCO_2_ slope > 44 with high risk (mortality at 1 year > 20%).

Several studies have addressed the topic of CPET as a tool to define PAH severity and prognosis; the main ones are summarized in Table 2.

The first study to highlight the prognostic value of CPET in PH was published in 2002. Wensel and colleagues [62], in a prospective population of 86 patients with primitive PH, 76 performing CPET both on a cycle ergometer or treadmill, found that peak VO_2_ and peak systolic blood pressure (SBP) were independent and highly accurate predictors of survival. Furthermore, apart from serum uric acid levels and peak diastolic blood pressure, all other clinical and hemodynamic variables measured in the study (which were predictive of survival in univariate analysis with good prognostic accuracy but failed to show independent prognostic value in multivariable analysis) did not provide additional prognostic information. It must be underlined that the V_E_/VCO_2_ slope was excluded from multivariate analysis, being calculated only in a few patients, albeit it was strongly predictive of survival in univariate analysis. Regardless of V_E_/VCO_2_ slope, excellent risk stratification could be obtained using the cut-off values of peak SBP and peak VO_2_ calculated by ROC curves: a peak SBP ≤ 120 mmHg and a peak VO_2_ ≤ 10.4 mL/kg/min were both found to be independent risk factors.

A subsequent retrospective analysis on 127 patients with PAH and CTEPH published by Groepenhoff and colleagues in 2008 [63] confirmed the prognostic value of CPET in PH. ROC curves from resting hemodynamic parameters and 6-min walking test (6MWT), and CPET showed that V_E_/VCO_2_ slope, V_E_/VCO_2_ nadir, V_E_/VCO_2_ peak, VO_2_ peak, the increase in O_2_ pulse from rest to peak exercise (∆O_2_ pulse), and 6MWT were accurate predictors of 4-year survival. However, from all CPET parameters, only ∆O_2_ pulse added a significant but modest prognostic value to the 6MWT.

The prognostic relevance of abnormalities in exercise ventilation was confirmed by Schwaiblmair et al. [64], who found significant differences between survivors and nonsurvivors in ventilatory equivalents for oxygen (V_E_/VO_2_) and for carbon dioxide (V_E_/VCO_2_) in 116 patients with both PAH and CTEPH during a follow-up of 24 months. Moreover, patients with peak VO_2_ ≤ 10.4 mL/min/kg had a 1.5-fold adverse events risk increase, V_E_/VCO_2_ ≥ 55 had a 7.8-fold, alveolar-arterial oxygen difference ≥55 mmHg had a 2.9-fold, and with V_E_/VCO_2_ slope ≥ 60 had a 5.8-fold increased risk of mortality.

In 2012, Deboeck et al. [65] demonstrated for the first time that exercise capacity predicts not only survival but also clinical stability as the time to clinical worsening (TTCW) in PAH, with better discrimination of exercise testing variables in IPAH than in associated PAH: in a population of 136 patients with PAH (85 idiopathic and 51 with associated conditions) having undergone CPET and 6MWT, a multivariable analysis showed that 6MWT and V_E_/CO_2_ at the AT predicted survival, while peak VO_2_ predicted TTCW. The ROC curve-derived cut-off values were 305 m for 6MWT, 54 for V_E_/VCO_2_ at the AT and 11.6 mL/kg/min for peak VO_2_. In the subgroup with associated PAH, no variable independently predicted either survival or clinical worsening.

Blumberg et al. [66] in 2013 showed for the first time a relationship between the hemodynamic response to exercise and functional capacity in patients with PH, also revealing that the ability to increase cardiac index (CI) on exercise affects survival. In a study population of 36 consecutive patients with both PAH or inoperable CTEPH undergone RHC at rest and during exercise, and CPET, exercise CI correlated with peak VO_2_ and was the only independent predictor of peak VO_2_ on multivariate analyses. Peak VO_2_ was the strongest predictor of survival, while among haemodynamic variables, only exercise CI and the slope of the pressure/flow relationship were significant prognostic indicators.

In 2013, Wensel et al. [67], analyzing 226 consecutive patients with idiopathic or familial PAH followed at seven specialized tertiary Centres, showed that on multivariate analysis % predicted of peak VO_2_, PVR, and increase in HR during exercise (ΔHR) were independent prognostic predictors. Peak VO_2_% of predicted allowed for accurate risk prediction with progressive deterioration of prognosis with decreasing peak VO_2_ (prognosis was favorable if peak VO_2_ ≥ 65% and acceptable if peak VO_2_ ≥ 46% predicted). Also, PVR yielded good risk stratification for the best and the worst quartile, with no significant survival differences between the middle quartiles. But most importantly, this study showed that dichotomizing by median peak VO_2_ and intra-group median PVR, patients with low peak VO_2_/higher PVR presented a worse 1-year survival compared to patients with low peak VO_2_/low PVR, high peak VO_2_/high PVR and high peak VO_2_/low PVR, underlining for the first time the complementary prognostic information from CPET and resting invasive haemodynamic data.

In the same year, Groepenhoff et al. [68] showed that, after PAH-specific therapy, survivors had a significantly greater increase in peak VO_2_ than nonsurvivors in a population of 65 idiopathic and heritable PAH patients, and this change in aerobic capacity was significantly related to changes in RV ejection fraction (RVEF) measured at cardiac magnetic resonance. Notably, survival analysis showed that baseline 6-min walking distance (6MWD), maximal HR, and V_E_/VCO_2_ slope were significant predictors of survival, whereas baseline VO_2_ and O_2_ pulse held no predictive value; on the other hand, treatment-associated changes in 6MWD, maximal HR, VO_2_, and O_2_ pulse predicted survival, whereas changes in V_E_/VCO_2_ slope did not. This study emphasized the importance of re-evaluating the prognostic parameters during follow-up.

Ferreira et al. [69] have demonstrated that measurements of excessive ventilation during exercise expressed both as a slope (ΔV_E_/ΔVCO_2_ to the respiratory compensation point and to exercise peak) and as a ratio (V_E_/VCO_2_ at the anaerobic threshold and at peak) in a population of idiopathic and associated PAH, maximize the usefulness of incremental CPET in the prognostic evaluation of PAH: multivariable regression analyses revealed that ΔV_E_/ΔVCO_2_ at peak < 55 and V_E_/VCO_2_ at peak < 57 were better related to prognosis than ΔV_E_/ΔVCO_2_ at the respiratory compensation point or at AT. In this study, however, the ΔVO_2_/Δwork rate > 5.5 mL/min/Watt was the only other independent prognostic index. According to a Kaplan-Meier survival analysis, 96.9% of patients showing ΔV_E_/ΔVCO_2_ at peak < 55 and ΔVO_2_/Δwork rate > 5.5 mL/min per W were free from a PAH-related event, while 74.7% with both parameters outside these ranges had a negative outcome.

The prognostic role of excessive exercise ventilation has also been confirmed in pediatric PAH: V_E_/VCO_2_ slope was significantly elevated in patients with poor outcome compared to patients who did not in a population of 76 children and young adults with a diagnosis of PH [70].

The role of the CPET in integrating the prognostic information provided by the parameters traditionally used in clinical practice in PAH has been further investigated by Badagliacca and colleagues. In a first study published in 2016 [71] on 102 consecutive naive patients with idiopathic PAH, Badagliacca et al. showed that echocardiography and CPET are important additions to RHC to assess disease severity and predict outcome: the multivariate analysis documented the increased power of a risk prediction model considering O_2_ pulse and echocardiographically-determined right ventricular fractional area change (RVFAC) compared to the traditional model including clinical, invasive haemodynamic and 6MWT variables without CPET parameters. Indeed, a combination of low RVFAC (<36.5%) and low peak O_2_ pulse (<8 mL/beat) identified a subgroup of patients at a particularly high risk of clinical deterioration, while patients with high RVFAC and high peak O_2_ pulse had a better prognosis compared with both patients with high RVFAC + low peak O_2_ pulse and low RVFAC + low peak O_2_ pulse, moreover, among patients with normal RVFAC, those with peak O_2_ pulse > 8 mL/beat showed a better prognosis compared to patients with low peak O_2_ pulse.

In a subsequent study of 2019 on a derivation cohort of 80 and a validation cohort of 80 PAH patients followed for 1 to 3 years of treatment [72], the combinations of baseline VO_2_ peak and change in CI during follow-up resulted in significant prognostication of low-risk patients with idiopathic, heritable, and drug-induced PAH. Indeed, while changes (∆) in WHO classification and CI and the absolute value of right atrial pressure (RAP) were independent predictors of clinical worsening at the first multivariate analysis, with the addition of CPET variables, peak VO_2_ and ∆CI independently improved the power of the prognostic model. ROC-derived cut-off values for ∆CI and VO_2_ peak were 0.40 L/min/m^2^ and 15.7 mL/kg/min (≥60% predicted value), respectively, and different combinations of these cut-offs defined four risk groups: the patients with high ∆CI + high VO_2_ peak combination showed the best event-free survival rates at 1, 2, and 3 years (100%, 100%, and 100%, respectively), while the groups with low ∆CI + low VO_2_ peak had the worst prognosis (survival rates 72%, 54%, and 33%); the event-free survival rates were 100%, 88%, and 71% for low ∆CI + high VO_2_ peak, and 80%, 54%, and 40% for high ∆CI + low VO_2_ peak.

In another study in 2022, Badagliacca and colleagues [73] found that combinations of VO_2_ peak and stroke volume index (SVI) may provide important information to further stratify intermediate-risk in idiopathic PAH patients. Two independent cohorts with idiopathic PAH at intermediate risk to develop (n = 124) and externally validate (n = 143) the prognostic model were evaluated. A risk score was constructed based on the β-coefficient of the cross-validated model, including the SVI and the peak VO_2_. Patients were grouped based on cut-off values of the risk score allowing the highest discrimination in the overall cohort. Group 1—score ≤ 2, with peak VO_2_ ≥ 14 mL/kg/min and SVI > 30 mL/m^2^, showed the best prognosis, as the event-free survival rates at 1, 2, and 3 years were 96%, 83%, and 79%, respectively; Group 2—score between 2 and 5, with peak VO_2_ between 9 and 14 mL/kg/min and SVI between 20 and 50 mL/m^2^, showed event-free survival rates of 82%, 67%, and 52, respectively; Group 3—score > 5, with peak VO_2_ peak < 10 mL/kg/min and SVI < 30 mL/m^2^, showed the worst prognosis (69%, 50%, and 41%, respectively).

Other CPET parameters have shown a prognostic value in PAH, including OUES [74], the persistence or development of an exercise-induced right-to-left shunt (individuated at CPET by an abrupt and sustained increase in P_ET_O_2_, with simultaneous and sustained decrease in P_ET_CO_2_, an abrupt and sustained increase in RER, V_E_/VO_2_ and V_E_/VCO_2_, and an associated decline in pulse oximetry) [75], and delayed post-exercise HR recovery response (<18 beats/min in the first minute post-exercise) [76].

Only a small study [77] assessed the role of gender on CPET parameters. In 21 male and 36 female incident IPAH patients who underwent both RHC and CPET, sex-specific CPET parameters resulted as predictors of poor outcomes, as decreased peak P_ET_CO_2_ in men and peak O_2_ pulse in women were associated with lower event-free survival.

CPET has also shown prognostic value in other PAH and PH groups.

In a study by Diller and colleagues [78], CPET was performed in 335 consecutive adult patients with congenital heart disease (ACHD), 40 non-congenital HF, and 23 healthy subjects. Peak VO_2_ was reduced in ACHD patients compared with healthy subjects of similar age, while no significant difference was found between ACHD and HF patients of the corresponding NYHA class. After a median follow-up of 10 months, on multivariable Cox analysis, peak VO_2_ predicted hospitalization or death and was related to the frequency and duration of hospitalization. In a subsequent study [79] on 560 ACHD patients of varying diagnoses and 50 healthy controls that had undergone CPET, cyanosis, with or without PAH, was the strongest predictor of abnormal V_E_/VCO_2_ slope, and the latter resulted as the most powerful univariate predictor of mortality in the noncyanotic group and the only independent predictor of mortality among exercise parameters on multivariate analysis, while in cyanotic patients, no parameter was predictive of death.

In a study on 151 retrospective patients with inoperable CTEPH [80], peak VO_2_ resulted as a significant predictor of survival when adjusting for age, gender, and PVR. However, peak VO_2_ failed as an independent prognostic factor in a stepwise multivariate model, including all variables significant in the univariate analysis.

A recent single-center observational [81] study on 89 patients with PH-LHD who had undergone RHC and CPET (mPAP > 20 mmHg and pulmonary artery wedge pressure ≥ 15 mmHg) showed that compared with survivors, nonsurvivors presented a significantly worse 6MWD, workload, exercise time, and peak VO_2_/kg with a trend of a lower OUES. Multivariate Cox regression revealed that the peak VO_2_/kg was significantly associated with all-cause death after adjusting for CpcPH/IpcPH.

## 7. Role of CPET in Clinical Trials on PAH

The role of CPET in clinical trials on PAH has been jeopardized by inconsistent results frequently observed. This was associated with the use of CPET by non-CPET experts.

Indeed, the STRIDE-1 trial on sitaxsentan treatment in PAH [82], the first trial using both 6MWT and CPET as endpoints, showed that sitaxsentan significantly improved the 6MWT distance but not peak VO_2_; moreover, the 6MWT and CPET parameters were not correlated, likely as a consequence of lack of sufficient expertise and accepted standardization methods in performing CPET [83]. This fact was recognized by Oudiz et al., who showed that the correlation between the 6MWT and CPET measurements improved significantly as the study progressed and as the technical skills at less-experienced sites improved [83].

In 2007, Oudiz et al. [84] studied 28 patients with idiopathic, associated with connective tissue disease and with corrected congenital heart disease PAH, all stable and receiving either conventional therapy or stable doses of prostanoids or endothelin receptor antagonists, 14 with and 14 without sildenafil treatment. CPET was performed before and after the addition of sildenafil: peak VO_2_, peak O_2_ pulse, V_E_/VCO_2_, and P_ET_CO_2_ improved after adding sildenafil, while control patients worsened.

Only recently, CPET has been reimplemented in clinical trials analyzing the effect of PAH treatment on CPET-derived parameters [85].

## 8. Future Perspectives

Several are the possible future utilizations of CPET in PH, including CPET combined with hemodynamic evaluation, both invasive and noninvasive, or with cardiac ultrasound imaging. In this regard, CPET has been used to investigate the effects of maximum incremental exercise on RV-arterial coupling in patients with exercise PH (ePH) and PAH undergoing maximum invasive CPET [86].

Another interesting application of CPET could concern the evaluation and the choice of therapeutic strategy in patients with mPAP between 20 and 25 mmHg: in fact, although the cut-off for the definition of pathology has been reduced to 20 mmHg in the latest guidelines [8], the specific treatment for PAH continues to be currently indicated only in patients with mPAP ≥ 25 mmHg and PVR > 3 WU, since there is still no evidence in patients with values below these cut-offs. Studies are needed to evaluate the impact of specific therapies in patients with the previously defined “borderline” PH, particularly in patients at risk of developing PH, such as SSc patients. CPET could provide important parameters to guide toward treatment or observation during follow-up in these subgroups of patients. Moreover, CPET may reduce the number of right heart catheterization procedures in patient follow-up. Finally, new drugs or new combinations of drugs must be analyzed by a multiparametric approach, including CPET. Unfortunately, the widespread use of CPET is still an unmet need, albeit nowadays, it is not possible to vision a PAH center without CPET capabilities.

## 9. Conclusions

In recent years, CPET has demonstrated through numerous evidences to be a valid tool in the management of patients with PH, providing insight into the pathophysiological mechanisms underlying the symptoms, supporting early detection and differential diagnosis, helping in prognostic stratification and in the follow-up, both of idiopathic and associated PAH, as well as in PH groups other than Group 1.

The ESC/ERS guidelines on PH have recognized and emphasized the value of CPET in this context; moreover, there are several interesting applications to further study.

It is, therefore, essential that an accurate knowledge of CPET and an adequate standardization of its execution are increasingly widespread in pulmonary hypertension centers. Indeed, it would nowadays be desirable that a PAH expert center try to gain a satisficing CPET capability.

## Figures and Tables

**Table 1:** Main studies investigating the role of cardiopulmonary exercise test in pulmonary hypertension groups of clinical classification other than Group 1.

Reference	Patients	Main Results
McCabe C et al., *Thromb. Res*. **2013**, *132*, 659–665 [54]	15 CTEPH 15 CTED	- Peak exercise VD/VT and A-a O_2_ gradient, HR/VO_2_ slope, V_E_/VCO_2_ slope, V_E_/VCO_2_ at the AT and P_ET_CO_2_ at AT discriminate between CTED and CTEPH groups. - VD/VT shows good correlation with mPAP. - In multivariate analysis, only VD/VT retains a significant predictive effect. - Peak exercise VD/VT > 45% has a sensitivity of 92% and specificity of 83% in predicting a diagnosis of CTEPH. - A peak exercise A-a O_2_ gradient > 32 mmHg has a sensitivity of 92% and specificity of 67%.
Guazzi et al., *J. Card. Fail.* **2013**, *19*, 461–467 [39]	293 HF	- V_E_/VCO_2_ slope ≥ 36 is the best predictor of a sPAP ≥ 40 mm Hg. - Peak P_ET_CO_2_ ≤ 34 mm Hg and the presence of EOV add significant diagnostic value.
Caravita et al., *J. Heart Lung Transplant.* **2017**, *36*, 754–762 [43]	29 IpcPH 12 CpcPH 29Idiopathic/Heritable PAH	- Exercise-induced hyperventilation (high V_E_/VCO_2_ slope, low P_ET_CO_2_) is marked in PAH, intermediate in CpcPH, low in IpcPH, and correlates with DPG and PVR. - Prevalence of EOB decreases from IpcPH to CpcPH to PAH.
Hirashiki A et al., *Ann. Noninvasive. Electrocardiol.* **2016**, *21*, 263–271 [55]	90 DCM	- A cut-off of peak VO_2_% predicted of 52.5% is the best predictor of an mPAP ≥ 25 mmHg. - Peak VO_2_% predicted is the only significant independent predictor of PH in the multivariate analysis.
Boerrigter BG et al., *Chest* **2012**, *142*, 1166–1174 [56]	47 COPD	- Patients with moderate PH (mPAP 25–39 mmHg at RHC) present only a mild alteration on V_E_/VCO_2_ slope and P_ET_CO_2_ compared with patients with no PH (mPAP < 25 mmHg). - Patients with severe PH (mPAP > 40 mmHg) present a much higher V_E_/VCO_2_ slope and lower P_ET_CO_2_ compared to both moderate and no PH patients, a lower mixed venous O_2_ saturation, preserved breathing reserve, and absence of CO_2_ increase during exercise.
van der Plas MN et al., *Respirology* **2014**, *19*, 269–275 [57]	38 IPF	- V_E_/VCO_2_ > 45 at AT significantly differentiates patients with and without PH (estimated sPAP ≥ 40 mmHg). - Patients with VE/VCO_2_ > 45 at the AT have a significantly lower survival compared to patients with V_E_/VCO_2_ ≤ 45. - sPAP does not allow survival prediction.
Gläser S et al., *PLoS ONE* **2013**, *8*, e65643 [58]	135 IPF	- Peak VO_2_ and V_E_/VCO_2_ slope differ significantly between IPF patients with and without PH. - Survival is best predicted by the presence of PH, followed by peak VO_2_.
Westhoff M et al., *Respiration* **2021**, *100*, 395–403 [59]	41 CPFE	- Peak A-a O_2_ gradient, V_E_/VCO_2_ slope, peak P(a-_ET_)CO_2_, and peak VO_2_ have high sensitivity, and when combined, a high specificity to detect PH.

A-a O_2_ gradient: alveolar-arterial oxygen gradient; AT: anaerobic threshold; CO_2_: carbon dioxide; COPD: chronic obstructive pulmonary disease; CpcPH: combined post-capillary pulmonary hypertension; CPFE: combined pulmonary fibrosis and emphysema; CTED: chronic thromboembolic disease; CTEPH: chronic thromboembolic pulmonary hypertension; DCM: dilated cardiomyopathy; DPG: diastolic pulmonary gradient; EOB: exercise oscillatory breathing; EOV: exercise oscillatory ventilation; HF: heart failure; HR: heart rate; IpcPH: isolated post-capillary pulmonary hypertension; IPF: idiopathic pulmonary fibrosis; mPAP: mean pulmonary arterial pressure; sPAP: systolic pulmonary arterial pressure; O_2_: oxygen; P(a-_ET_)CO_2:_ arterial to end-tidal carbon dioxide tension difference; PAH: pulmonary arterial hypertension; P_ET_CO_2_: end-tidal pressure of carbon dioxide; PH: pulmonary hypertension; PVR: pulmonary vascular resistance; RHC: right heart catheterization; VD/VT: dead space/tidal volume ratio; V_E_/VCO_2_: ventilatory equivalent for carbon dioxide; V_E_/VCO_2_ slope: ventilation/carbon dioxide production relationship slope; VO_2_: oxygen consumption.

**Table 2:** Main studies investigating the prognostic role of cardiopulmonary exercise test in pulmonary hypertension.

Reference	Patients	Main Results
Wensel et al., *Circulation* **2002**, *106*, 319–324 [62]	76 PPH	- Peak VO_2_ and peak SBP are independent and highly accurate predictors of survival. - A peak SBP ≤ 120 mmHg and a peak VO_2_ ≤ 10.4 mL/kg/min are independent risk factors.
Groepenhoff H et al., *Med. Sci. Sports Exerc.* **2008**, *40*, 1725–1732 [63]	127 PAH and CTEPH	- V_E_/VCO_2_ slope, V_E_/VCO_2_ nadir, V_E_/VCO_2_ peak, VO_2_ peak, ∆O_2_ pulse, and 6MWT are accurate predictors of 4 yr survival. - ∆O_2_ pulse adds a significant but modest prognostic value to 6MWT.
Schwaiblmair et al., *BMC Pulm. Med.* **2012**, *12*, 23 [64]	116 PAH and CTEPH	- Significant differences between survivors and nonsurvivors in V_E_/VO_2_ and V_E_/VCO_2_ during 24-month FU. - Patients with peak VO_2_ ≤ 10.4 mL/min/kg have a 1.5-fold, V_E_/VCO_2_ ≥ 55 a 7.8-fold, ∆A-a O_2_ ≥ 55 mmHg a 2.9-fold and V_E_/VCO_2_ slope ≥ 60 a 5.8-fold increased risk of mortality in the next 24 months.
Deboeck et al., *Eur. Respir. J.* **2012**, *40*, 1410–1419 [65]	136 PAH (idiopathic and associated)	- 6MWT and V_E_/CO_2_ at AT predict survival. - Peak VO_2_ predicts TTCW. - ROC curve-derived cut-off values: 305 m for 6MWT, 54 for V_E_/VCO_2_ at the AT, and 11.6 mL/kg/min for peak VO_2_. - In the subgroup with associated PAH, no variable independently predicts survival or CW.
Blumberg et al., *Eur. J. Heart Fail.* **2013**, *15*, 771–775 [66]	36 PAH and CTEPH	- Exercise CI correlates with peak VO_2_ and is the only independent predictor of peak VO_2_ on multivariate analysis. - Peak VO_2_ is the strongest predictor of survival. - Exercise CI and the slope of the pressure/flow relationship are significant prognostic indicators.
Wensel et al., *Int. J. Cardiol.* **2013**, *167*, 1193–1198 [67]	226 idiopathic and familial PAH	- % predicted of peak VO_2_, PVR, and ΔHR are independent prognostic on multivariate analysis. - Prognosis is favorable if peak VO_2_ ≥ 65% and acceptable if peak VO_2_ ≥ 46% predicted. - PVR yields good risk stratification for the best and the worst quartile, with no significant survival differences between the middle quartiles. - Patients with low peak VO_2_/higher PVR (median value) present a worse 1-year survival compared to patients with low peak VO_2_/low PVR, high peak VO_2_/high PVR, and high peak VO_2_/low PVR.
Groepenhoff H. et al., *PloS ONE* **2013**, *8*, e72013 [68]	65 idiopathic and heritable PAH	- Survivors have a significantly greater change in peak VO_2_ than nonsurvivors after specific treatment. - Changes in peak VO_2_ are significantly related to changes in RVEF. - Baseline 6MWT, maximal HR, and V_E_/VCO_2_ slope are significant predictors of survival. - Treatment-associated changes in 6MWT, maximal HR, VO_2_, and O_2_ pulse predict survival.
Ferreira EV et al., *Eur. J. Prev. Cardiol.* **2014**, *21*, 1409–1419 [69]	84 idiopathic and associated PAH	- ΔV_E_/ΔVCO_2_ at peak < 55 and V_E_/VCO_2_ at peak < 57 are better related to prognosis than ΔV_E_/ΔVCO_2_ at the respiratory compensation point or at AT. - ΔVO_2_/Δwork rate > 5.5 mL/min per W is the only other independent prognostic index. - 96.9% of patients with ΔV_E_/ΔVCO_2_ at peak < 55 and ΔVO_2_/Δwork rate > 5.5 mL/min per W are free from a PAH-related event, 74.7% with both parameters outside these ranges had a negative outcome.
Rausch CM et al., *Int. J. Cardiol.* **2013**, *169*, 445–448 [70]	76 pediatric PH	- V_E_/VCO_2_ slope is significantly elevated in patients with poor outcome.
Badagliacca R et al., *Chest* **2016**, *150*, 1313–1322 [71]	102 IPAH	- At multivariate analysis increased power of a risk prediction model considering O_2_ pulse and RVFAC compared to the traditional model, including clinical, invasive haemodynamic, and 6MWT variables without CPET parameters. - A combination of low RVFAC and low O_2_ pulse identifies a subgroup of patients at a particularly high risk of clinical deterioration.
Badagliacca R et al., *J. Heart Lung. Transplant.* **2019**, *38*, 306–314 [72]	80 IPAH, HPAH, drug-induced PAH 80 HPAH, drug-induced PAH (validation cohort)	- Changes in WHO class, CI, and RAP are independent predictors of CW at the first multivariate analysis. - With the addition of CPET variables, peak VO_2_ and ∆CI independently improve the power of the prognostic model. - ROC-derived cut-off values for ∆CI and VO_2_ peak are 0.40 L/min/m^2^ and 15.7 mL/kg/min, respectively. - The event-free survival rates at 1, 2, and 3 years are 100%, 100%, and 100%, respectively, for the high ∆CI + high VO_2_ peak combination; 100%, 88%, and 71% for low ∆CI + high VO_2_ peak; 80%, 54%, and 40% for high ∆CI + low VO_2_ peak; and 72%, 54%, and 33% for low ∆CI + low VO_2_ peak.
Badagliacca R et al., *J. Heart Lung Transplant.* **2022**, *41*, 780–790 [73]	124 IPAH 143 IPAH (validation cohort)	- A risk score based on the β-coefficient of the cross-validated model, including the SVI and the peak VO_2_. - Four patients groups based on cut-off values of the risk score: Group 1, score ≤ 2 with VO_2_ peak ≥ 14 mL/kg/min and SVI > 30 mL/m^2^; Group 2, score between 2 and 5 with VO_2_ peak between 9 and 14 mL/kg/min, and SVI between 20 and 50 mL/m^2^; Group 3, score > 5 with VO_2_ peak < 10 mL/kg/min and SVI < 30 mL/m^2^. - The event-free survival rates at 1, 2, and 3 years, are 96%, 83%, and 79% for Group 1; 82%, 67%, and 52% for Group 2; 69%, 50%, and 41% for Group 3, respectively.
Tang Y et al., *J. Am. Heart Assoc.* **2017**, *6*, e005037 [74]	210 PAH	- OUES, OUESI, peak VO_2_, V_E_/VCO_2_ slope, peak SBP, HR recovery, PVR, CI, NTproBNP, and WHO class are predictive of CW in univariate analysis. - Patients with OUESI ≤ 0.52 m^2^ have a worse 5-year survival rate than patients with OUESI > 0.52 m^2^.
Oudiz RJ et al., *Am. J. Cardiol.* **2010**, *105*, 1186–1191 [75]	103 PAH	- The persistence or development of an exercise-induced right-to-left shunt strongly predicts death or transplantation at multivariate analsysis.
Ramos R.P. et al., *Am. Heart J.* **2012**, 163, 580–588 [76]	72 PAH	- Compared with patients with HRR_1min_ ≤ 18, patients with HRR_1min_ > 18 have better NYHA, resting hemodynamics, and 6MWT. - 6MWT is the single independent predictor of HRR_1min_ ≤ 18. - On a multiple regression analysis considering only CPET-independent variables, HRR_1min_ ≤ 18 is the single predictor of mortality.
Yuan P. et al., *Hypertens Res.* **2017**, *40*, 868–875 [77]	57 (21 male/36female) IPAH	- Decreased peak P_ET_CO_2_ in men and peak O_2_ pulse in women are associated with lower event-free survival.
Diller G.P. et al., *Circulation* **2005**, *112*, 828–835 [78]	335 ACHD 40 non-congenital HF 23 healthy subjects	- Peak VO_2_ is reduced in ACHD compared with healthy subjects. - No significant difference between ACHD and HF. - After a median follow-up of 10 months, peak VO_2_ predicts hospitalization or death, and is related to the frequency and duration of hospitalization.
Dimopoulos K. et al., *Circulation* **2006**, *113*, 2796–2802 [79]	560 ACHD 50 healthy subjects	- Cyanosis, with or without PAH, is the strongest predictor of abnormal V_E_/VCO_2_ slope. - V_E_/VCO_2_ slope is the most powerful univariate predictor of mortality in the noncyanotic group and the only independent predictor of mortality among exercise parameters on multivariate analysis. - In cyanotic patients, no parameter was predictive of death.
Richter M.J. et al., *Clin. Respir. J.* **2017**, *11*, 682–690 [80]	151 CTEPH	- Peak VO_2_ is a significant predictor of survival when adjusting for age, gender, and PVR. - Peak VO_2_ fails as an independent prognostic factor in a stepwise multivariate model, including all variables significant in the univariate analysis.
Zhong X.J. et al., *BMC* *Cardiovasc*, *Disord.* **2002**, *22*, 137 [81]	89 PH-LHD	- Nonsurvivors have significantly worse 6MWT, workload, exercise time and peak VO_2_/kg with a trend of a lower OUES compared to survivors. - Peak VO_2_/kg is significantly associated with all-cause death after adjusting for CpcPH/IpcPH at multivariate analysis.

6MWT: 6-min walk test; ΔHR: increase in heart rate during exercise; ∆O_2_ pulse: increase in O_2_ pulse from rest to peak exercise; ACHD: adult patients with congenital heart disease; AT: anaerobic threshold; CPFE: combined pulmonary fibrosis and emphysema; COPD: chronic obstructive pulmonary disease; CpcPH: combined post-capillary pulmonary hypertension; CI: cardiac index; CTED: chronic thromboembolic disease; CTEPH: chronic thromboembolic pulmonary hypertension; CW: clinical worsening; DCM: dilated cardiomyopathy; FU: follow-; HF: heart failure; HR: heart rate; HRR_1min_: heart rate recovery in 1 min; IpcPH: isolated post-capillary pulmonary hypertension; IPAH: idiopathic pulmonary arterial hypertension; IPF: idiopathic pulmonary fibrosis; NYHA: New York Heart Association; NTproBNP: N-terminal pro-brain natriuretic peptide; OUES: oxygen uptake efficiency slope; OUESI: OUES/body surface area; PH: pulmonary hypertension; PH-LHD: pulmonary hypertension associated to left heart disease; PVR: pulmonary vascular resistance; ROC: receiver operating characteristic curve RVEF: right ventricular ejection fraction; SBP: systolic blood pressure; TTCW: time to clinical worsening; V_E_/VCO_2_: ventilatory equivalent for carbon dioxide; V_E_/VCO_2_ slope: ventilation/carbon dioxide production relationship slope; VO_2_: oxygen consumption; WHO: World Health Organization.
